# Advances in EBSD-based approaches for quantifying slip activity in deformed Mg alloys

**DOI:** 10.1186/s42649-026-00124-y

**Published:** 2026-02-12

**Authors:** Hafiz Muhammad Rehan Tariq, Tea-Sung Jun

**Affiliations:** 1 Department of Mechanical Engineering, Incheon National University, Incheon, 22012 Republic of Korea; 2 Research Institute for Engineering and Technology, Incheon National University, Incheon, 22012 Republic of Korea

**Keywords:** Mg alloy, Dislocation Slip, Electron Backscattered Diffraction, Crystal Orientation, Schmid Factor Analysis, In grain misorientation Axis (IGMA) analysis

## Abstract

Plastic deformation in Mg alloys requires a full understanding of slip activity and intergranular interactions, which determine mechanical behavior and strain localization. Electron backscatter diffraction (EBSD) has emerged as a versatile technique to map crystallographic orientations, slip systems, and lattice rotations, permitting the systematic analysis of deformation mechanisms across polycrystalline aggregates. The coupling of EBSD with metrics including Schmid factors, ingrain misorientation axes, and slip-transfer criteria permits a quantitative assessment of slip compatibility and the role of grain boundaries in strain accommodation. Limitations related to conventional 2D surface characterization create a growing need for novel three-dimensional techniques that can accurately represent grain boundary geometry as well as complex intergranular deformation pathways. A focused review of such methodologies will compile current knowledge on these methods and their capabilities and limitations, guiding future investigations toward a deeper understanding of microstructure-mechanics relationships in Mg alloys.

## Introduction

Magnesium (Mg) and its alloys are among the lightest structural metallic materials, offering an exceptional combination of low density and high specific strength, which makes them attractive for applications where weight reduction is critical, such as in the automotive, aerospace, and electronics industries (Chaudry et al. 2024a, 2025; Kannan et al. 2025a; Tariq et al. 2025a, 2025b). However, despite these advantageous characteristics, Mg alloys exhibit poor formability and limited ductility at ambient temperatures (Hafiz Muhammad Rehan et al. 2025; Tariq et al. 2024a). This inherent limitation originates primarily from their hexagonal close-packed (HCP) crystal structure, which provides a restricted number of active deformation modes compared to face-centered cubic (FCC) or body-centered cubic (BCC) metallic systems (Kannan et al. 2025b; Ishtiaq et al. 2025a; Siddique et al. 2025a, 2025b; Dilshodbek et al. 2023; Yusupov et al. 2025). The HCP crystal structure of Mg allows only a limited number of easily activated slip systems at ambient temperatures. The predominant mode of plastic deformation in Mg is basal slip, which occurs along the {0001} plane. This system requires the lowest critical resolved shear stress (CRSS) and accommodates shear parallel to the basal plane (Chaudry et al. 2024b, 2019). However, basal slip alone cannot produce homogeneous plastic deformation, since it provides only two independent slip systems, which is insufficient to satisfy the von Mises criterion, which suggests at least five independent slip systems for homogeneous plastic flow in polycrystalline materials (Malik et al. 2025; Chaudry et al. 2022a). As a result, Mg alloys tend to localize strain and fracture prematurely, resulting in poor ductility. In addition to basal slip, Mg crystals can theoretically deform by non-basal slip on prismatic < a > and pyramidal {10–11} and {11–22} planes. These non-basal slip systems can accommodate deformation components along the crystallographic c-axis; however, they require substantially higher CRSS values for activation compared with basal slip. The hierarchy of CRSS among these slip systems governs the relative activity of each mechanism (Chaudry et al. 2022b). Atomistic simulations by Wu et al. revealed that dislocations in the pyramidal {11–22} < c + a > system tend to dissociate into partials on basal planes, forming sessile dislocation segments that impede further glide (Wu and Curtin 2015). This dissociation reduces dislocation mobility and aggravates strain hardening, further restricting the activation of c-axis deformation. The deformation behavior of Mg alloys is also strongly influenced by their crystallographic texture, which evolves during primary thermomechanical processes such as rolling or extrusion (Tariq et al. 2024b). These processes typically produce a strong basal texture, characterized by the alignment of the c-axis nearly parallel to the sheet normal direction. In such textures, the basal slip system exhibits a low Schmid factor, leading to a low resolved shear stress (Malik 2025). As a result, dislocation glide on basal planes is hindered, and the alloy exhibits poor ductility when loaded along the rolling direction. In contrast, weak or tilted basal textures, where many grains have c-axis inclined away from the ND, display higher Schmid factors (up to ~ 0.5) and improved ductility due to easier activation of basal and non-basal slip systems (Lee et al. 2025).

Therefore, identification of active slip systems in Mg alloys is essential for understanding their deformation mechanisms. Traditionally, transmission electron microscopy (TEM) combined with the Burgers vector analysis method has been employed to determine the nature of dislocations and, consequently, the operative slip systems (Ishtiaq et al. 2025b, 2025c). Although this technique provides high spatial resolution and precise crystallographic information, its practical application is constrained by the inherently small observation area and the extensive time required for data collection. To overcome these limitations, numerous investigations have employed electron backscatter diffraction (EBSD) based approaches to identify and analyze slip activity in deformed polycrystalline materials (Britton and Hickey 2018). Leveraging the crystallographic orientation data provided by EBSD, various analytical strategies have been developed to extract information related to active slip systems and local plastic deformation. These methods enable the inference of slip activity directly from orientation gradients, lattice rotations, and surface trace geometry, offering a more comprehensive understanding of deformation behavior across multiple grains.

The present review aims to systematically consolidate and evaluate all EBSD-based methodologies reported to date for determining slip activity in deformed polycrystals, with a particular emphasis on their capabilities and limitations in Mg alloys.

## Fundamentals of slip activity and orientation analysis by EBSD

**Fig. 1:**
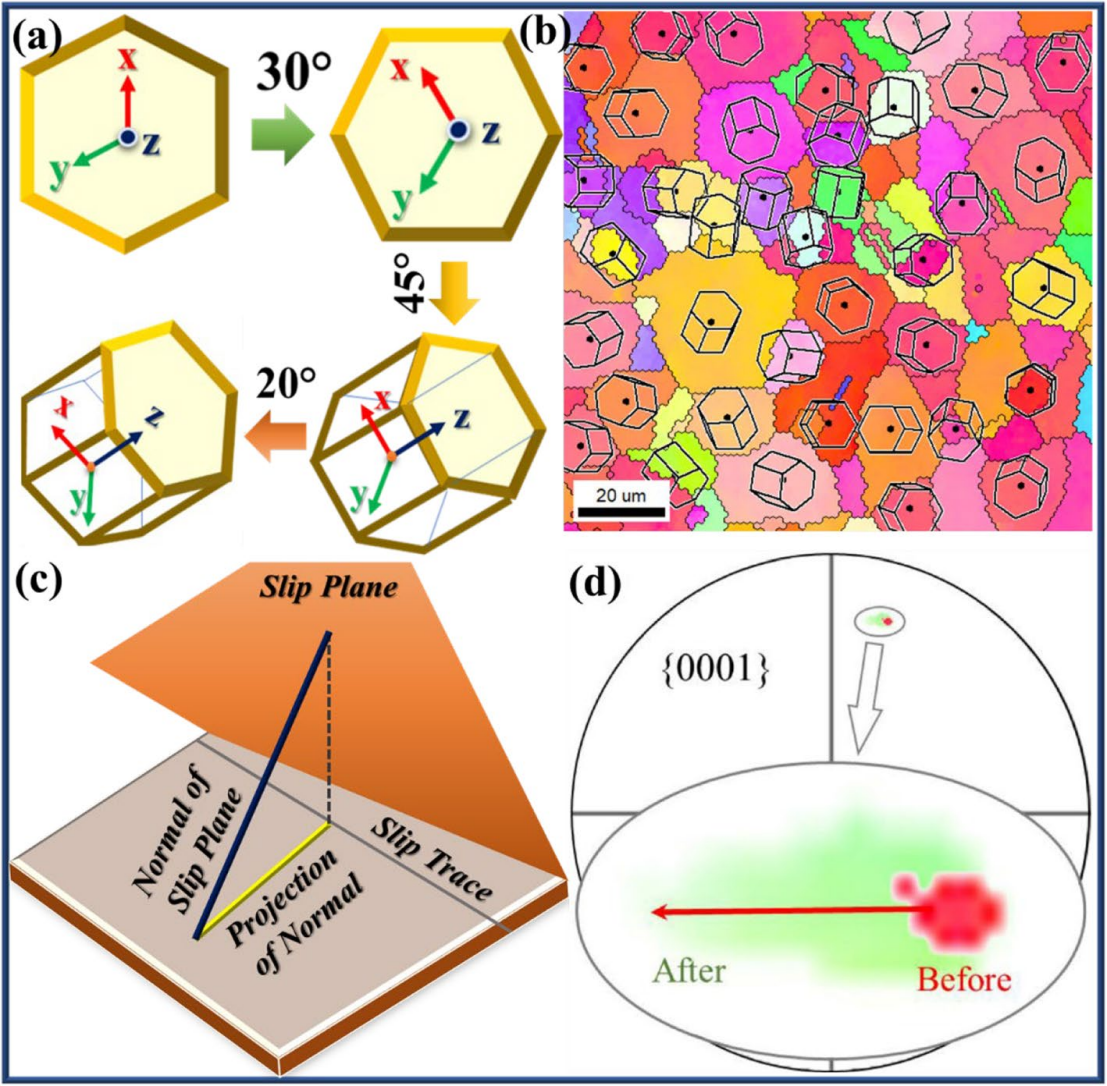
**a** Illustration of a crystal initially oriented at Euler angles (0°, 0°, 0°) and subsequently rotated to (30°, 45°, 20°) (Li et al. 2022). **b** EBSD-derived Inverse Pole Figure (IPF) map of the AZX311 Mg alloy, revealing grains with diverse crystallographic orientations. **c** Conceptual diagram illustrating the geometric relationship between the slip plane, its normal, the projection of that normal on the sample surface, and the resulting slip trace; the projected normal is perpendicular to the slip trace. **d** Experimental {0001} pole figure of grain recorded before (red) and after (green) deformation, with the principal elongation direction of the grain trace indicated by a red arrow (Yang et al. 2022)

The orientation of the grain can be described by Euler angles (φ1, Φ, φ2) using the OIM software. Figure 1a shows an example of a crystal with Euler angles (0°, 0°, 0°) and subsequent rotation to the Euler angles (30°, 45°, 20°). The crystal with (0°, 0°, 0°) was rotated by 30°, 45°, and 20° in the counterclockwise direction around the Z-axis, X-axis, and Z-axis, respectively (Li et al. 2022). The angle between the X- and Y-axis was 120°, and the Z-axis was perpendicular to the X- and Y-axis and parallel to the c-axis of the Mg crystal. As the crystal was rotated, its XYZ coordinate system was rotated simultaneously. A four-axis coordinate system is typically used to represent the crystal planes and crystal directions in hexagonal structures, e.g., {hkil}- < uvtw > respectively. To facilitate the following calculations, a cartesian coordinate system was introduced (as shown in Fig. 1a). The rotation axis of the X- and Z-axis are denoted as < 010 > and < 001 > in the Cartesian coordinate system.

The analysis of slip activity in polycrystalline materials, particularly Mg alloys, relies heavily on crystallographic orientation data obtained through EBSD. Grain orientation is typically described using Euler angles (φ1, Φ, φ2), which specify the rotation of a crystal with respect to the sample reference frame. Orientation Imaging Microscopy (OIM) software is commonly used to determine these angles and visualize grain orientations. Euler angles provide a standardized method for describing the orientation of crystal lattices (Schwartz et al. 2009). An initial reference orientation (0°, 0°, 0°) signifies an unrotated lattice, while subsequent rotations around a specific axis produce new orientations such as (30°, 45°, 20°). In hexagonal close-packed (HCP) systems like Mg alloys, the crystallographic axis (X, Y, Z) corresponds to the < 010 >, < 120 >, and < 001 > directions, with the Z-axis aligned parallel to the c-axis. A Cartesian coordinate system is often used for further calculations, allowing for straightforward analysis of slip systems and traces (Ganeshan et al. 2009).

EBSD-derived inverse pole figure (IPF) maps reveal the diversity of grain orientations within an alloy, such as shown in the IPF map of undeformed AZX311 Mg alloy (Fig. 1b), where each color denotes a unique orientation. These maps are fundamental for assessing texture, grain boundary character, and the probability of slip or twinning events under mechanical loading (Ganeshan et al. 2009; Tariq et al. 2024c). Such orientation information enables identification of active slip systems during deformation, with direct implications for mechanical behavior and anisotropic response (Parkin and Birosca 2021). Slip activity in HCP alloys is governed by the geometric relationship between slip planes and their normal. The projection of the slip-plane normal onto the sample surface is perpendicular to the corresponding slip trace. Regardless of slip direction within a basal (or other) plane, the slip trace remains fixed for a given grain orientation under the assumption of dominant slip-system activity (Sarebanzadeh et al. 2023). Using grain orientation data, the normal to any slip or twin plane can be calculated, and possible slip traces on the specimen surface can be predicted. These geometric relationships underpin methods for identifying active deformation mechanisms based on EBSD data. ​Pole figures constructed from EBSD data display the distribution of specific crystallographic planes, such as {0001}, before and after deformation. Changes in pole distribution, evidenced by shifts or elongation, are associated with grain rotation, slip activity, or twinning (Yang et al. 2022). The direction and magnitude of trace elongation on the pole figure reflect underlying deformation processes and highlight the evolution of crystallographic texture under applied stress. ​

Overall, EBSD orientation analysis provides a powerful, quantitative means for correlating microstructural features and enables researchers to systematically explore and model the role of grain orientation in deformation mechanisms and material performance.

## Schmid factor analysis

**Fig. 2:**
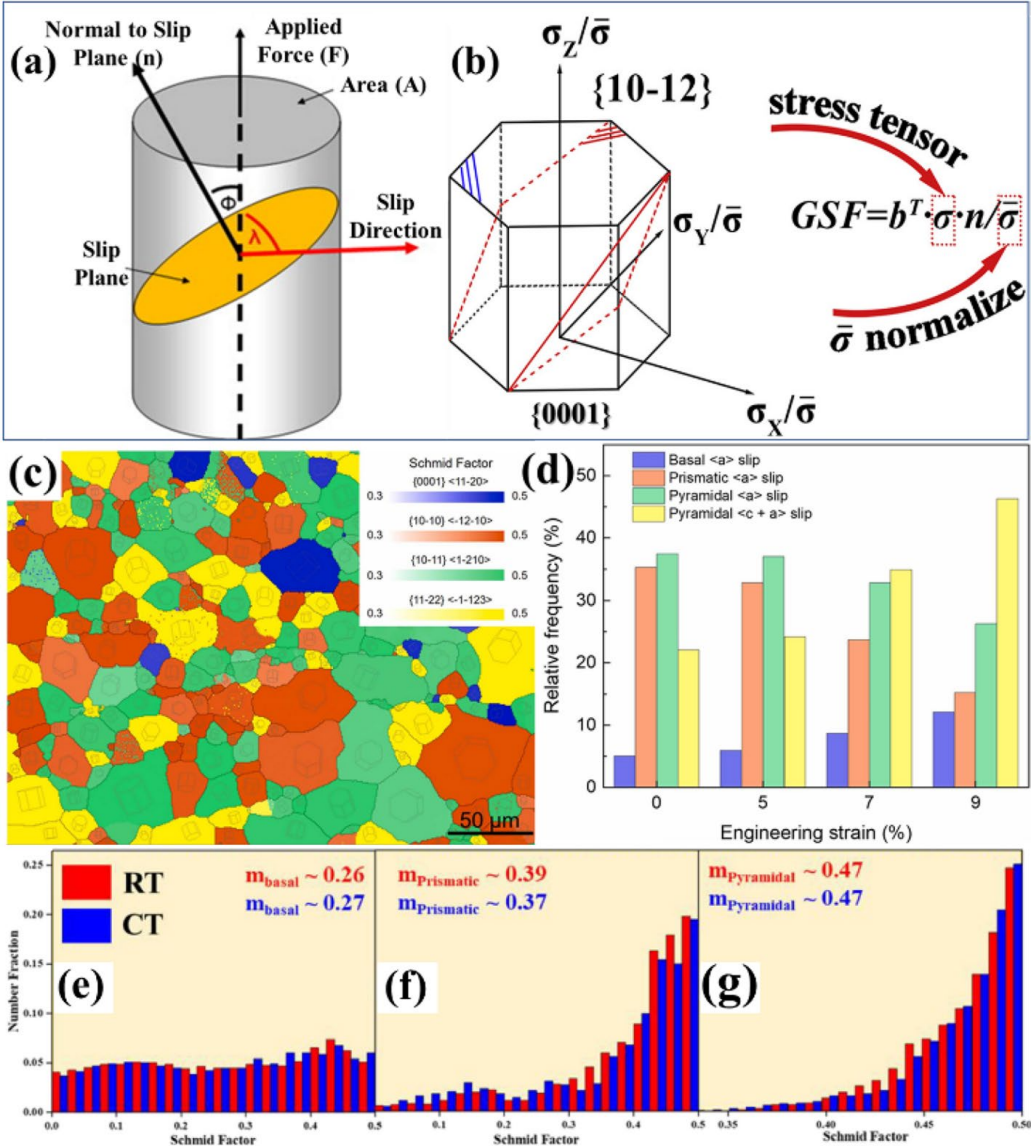
**a** Schematic representation of the orientation between applied load, the slip plane and the slip direction in a single crystal (Breach and Wulff 2010) (**b**) Generalized Schmid factor (GSF) schematic showing the normalized stress components in an HCP crystal and the tensor form for evaluating resolved shear stress under 3D loading (Xia et al. 2019) (**c, d**) Observation of SF distribution during uniaxial compression along the ED: **c** map showing the favorable slip system of each grain, **d** relative frequencies of various slip systems under different strains in AZ31 Mg alloy (Xu et al. 2025) (**e–g**) Histograms representing the Schmid Factor distributions for (**e**) basal, (**f**) prismatic, and (**g**) pyramidal slip systems, with corresponding average Schmid factors (m values) indicated on each graph of deformed AZX311 Mg alloy at room and cryogenic temperatures (Tariq et al. 2024d)

The initiation of plastic deformation in crystalline solids is governed by the crystallographic orientation of the active slip systems relative to the direction of the applied external load. This orientation dependence is quantitatively expressed by the Schmid factor, which determines the magnitude of the resolved shear stress acting on a specific slip system under a given applied stress state. As shown schematically in Fig. 2(a), the deformation of a single crystal under uniaxial loading can be resolved into shear components acting along the preferred slip direction within the active slip plane. The geometric relationship between the applied stress (σ) and the resolved shear stress (τ_s​_) is defined by the angles φ and λ, where φ represents the angle between the normal to the slip plane and the loading direction, and λ denotes the angle between the slip direction and the same loading axis (Wu et al. 2008). The resolved shear stress is thus expressed as;

1 $${\boldsymbol\uptau}_{\mathbf s}\boldsymbol{=\sigma}\mathbf{m}$$

where;

2 $$\mathbf m\boldsymbol=\mathbf{cos}\mathbf{\varPhi}\mathbf{cos}\mathbf{\lambda}$$

This dimensionless parameter quantifies the efficiency with which the externally applied normal stress is transformed into the shear stress that drives dislocation motion. The following equation can further estimate these cosine functions (Nan et al. 2012);

3 $$\mathbf{cos}\;\mathbf\upvarphi\boldsymbol\;\mathbf{\left(\uplambda\right)}\boldsymbol\;\boldsymbol=\boldsymbol\;\frac{{{\boldsymbol{\upsigma}}1\mathbf{x}}^{{\boldsymbol{m}}}+{{\boldsymbol{\upsigma}}2\mathbf{x}}^{{\boldsymbol{m}}}+{{\boldsymbol{\upsigma}}3\mathbf{x}}^{{\boldsymbol{m}}}}{{[{{\boldsymbol{\upsigma}}1}^{2}+{{\boldsymbol{\upsigma}}2}^{2}+{{\boldsymbol{\upsigma}}3}^{2}]}^{1/2}{[{\mathbf{x}}^{{\boldsymbol{m}}2}+{\mathbf{y}}^{{\boldsymbol{m}}2}+{\mathbf{z}}^{{\boldsymbol{m}}2}]}^{\boldsymbol1/2}}$$

Here *x*^m^, *y*^m^ and *z*^m^ denote the slip-plane normal or slip direction after the crystal orientation has been rotated into the sample reference frame. Unlike dislocation slip, which can operate in both positive and negative shear directions, twinning accommodates shear in only a single, specific direction (Liu et al. 2025). Consequently, the Schmid factor (m) for dislocation slip can be evaluated using its absolute value.

Crystals possessing a higher Schmid factor require lower applied stresses to reach the CRSS necessary for the onset of slip, and therefore, deform plastically at lower loads. Conversely, crystals oriented with lower Schmid factors exhibit greater resistance to plastic flow and may instead experience cleavage or brittle fracture if the applied stress exceeds the fracture threshold before reaching the CRSS (Shin et al. 2023). The maximum theoretical value of the Schmid factor, m = 0.5, occurs when both φ and λ are 45°, corresponding to the optimal orientation for slip activation. This geometrical condition reflects the state in which the applied stress is most effectively resolved into shear on the slip plane, providing a fundamental link between crystallographic orientation and macroscopic mechanical response. Figure 2(b) further extends the Schmid law framework to three-dimensional stress states, depicting a generalized relationship between the applied stress tensor and the crystallographic orientation of the slip system in a hexagonal close-packed (HCP) lattice. The hexagon represents normalized stress components (σ_x_/$\-{\sigma }$, σ_y_/$\-{\sigma }$, σ_z_/$\-{\sigma }$) that correspond to the principal stresses acting on the crystal. The generalized Schmid factor (GSF) can thus be mathematically expressed as GSF = b^*T*^ σ_n_/$\-{\sigma }$, where *b* is the Burgers vector representing the slip direction, *σ* is the stress tensor, and *n* is the unit normal vector to the slip plane. The normalization by $\-{\sigma }$ ensures dimensionless comparison across different loading conditions. This tensorial representation enables accurate determination of the resolved shear stress for complex loading paths, non-uniaxial stress states, or materials with anisotropic elastic and plastic properties—conditions commonly encountered in HCP metals such as Mg alloy (Zubelewicz 2023).

The implications of Schmid factor analysis extend well beyond theoretical mechanics. In practice, it provides a predictive framework for identifying slip initiation sites, analyzing anisotropic yielding, and interpreting deformation textures in both experimental and computational studies. For instance, orientation imaging microscopy (OIM) (applied on EBSD data) routinely employs Schmid factor mapping to predict regions of preferential slip activation and to correlate local orientation distributions with macroscopic mechanical anisotropy. For example, Xu et al. reported that the distribution of favorable slip systems in the initial microstructure can be visualized by assigning each grain the slip system with the highest Schmid factor (Xu et al. 2025). As shown in Fig. 2c, four representative slip systems in Mg, basal < a >, prismatic < a >, pyramidal < a >, and pyramidal < c + a >, were evaluated for every grain in the undeformed AZ31 alloy. Each grain is color-coded according to the slip system with the highest SF, allowing the preferential deformation pathway of each grain to be mapped across the microstructure. This representation highlights that the initial texture strongly biases the material toward non-basal slip: grains favorable for prismatic and pyramidal slip are widely dominant, whereas grains with high SF for basal < a > slip are least frequent. Further statistical evolution of these slip preferences with increasing strain is presented in Fig. 2d. Although prismatic and pyramidal slip maintain relatively high activity during early deformation, the proportions of grains favoring basal < a > slip and pyramidal < c + a > slip increase markedly as compression progresses. Quantitatively, the fraction of grains oriented favorably for basal < a > slip rises from approximately 5.1% in the undeformed state to 12.1% at 9% strain. More notably, the proportion of grains with the highest SF for pyramidal < c + a > slip increases from 22.1% to 46.3% over the same strain interval. This trend indicates a progressive redistribution of deformation pathways, where increasing applied strain activates additional systems, particularly basal and < c + a > pyramidal slip, to accommodate the evolving strain state.

Similarly, Tariq et al. reported that the average Schmid factor distributions for basal, prismatic, and pyramidal slip systems reveal clear differences in slip activity for AZX311 Mg alloy samples deformed at both room and cryogenic temperatures. The basal slip systems exhibit relatively low m values of about 0.26 and 0.27, whereas prismatic slip systems show moderately higher values of approximately 0.39 and 0.37. Pyramidal slip systems display the highest Schmid factors, reaching roughly 0.47 in both temperature conditions. Because slip systems with Schmid factors approaching 0.5 are more easily activated, these results indicate that the AZX311 alloy preferentially engages prismatic and pyramidal slip regardless of deformation temperature (Tariq et al. 2024d).

The concept of the Schmid factor becomes considerably more complex in polycrystalline materials due to the interaction among differently oriented grains. In such systems, the macroscopic plastic deformation is not solely determined by the most favorably oriented grain but rather by the collective and cooperative yielding of an ensemble of grains (Liu et al. 2023). Under an applied load, grains that have a high Schmid factor are oriented in a way that makes it easy for slip to start, so they begin to plastically deform first. In contrast, grains with a low Schmid factor are not well aligned for slip and therefore stay mostly elastic and deform less at the beginning. For polycrystalline aggregates, the Schmid factor concept is generalized through the Taylor factor (M), which links the macroscopic flow stress (σ) to the critical shear stress (τ) via the relationship σ = *M*τ. The Taylor factor embodies the constraint imposed by the collective deformation of multiple grains with distinct orientations, effectively representing the reciprocal of the average Schmid factor across all active slip systems (Mecking et al. 1996). Since each grain in a polycrystal must deform compatibly with its neighbors, higher external stresses are typically required to achieve the same level of shear strain observed in single crystals.

## In-grain misorientation axis analysis

**Fig. 3:**
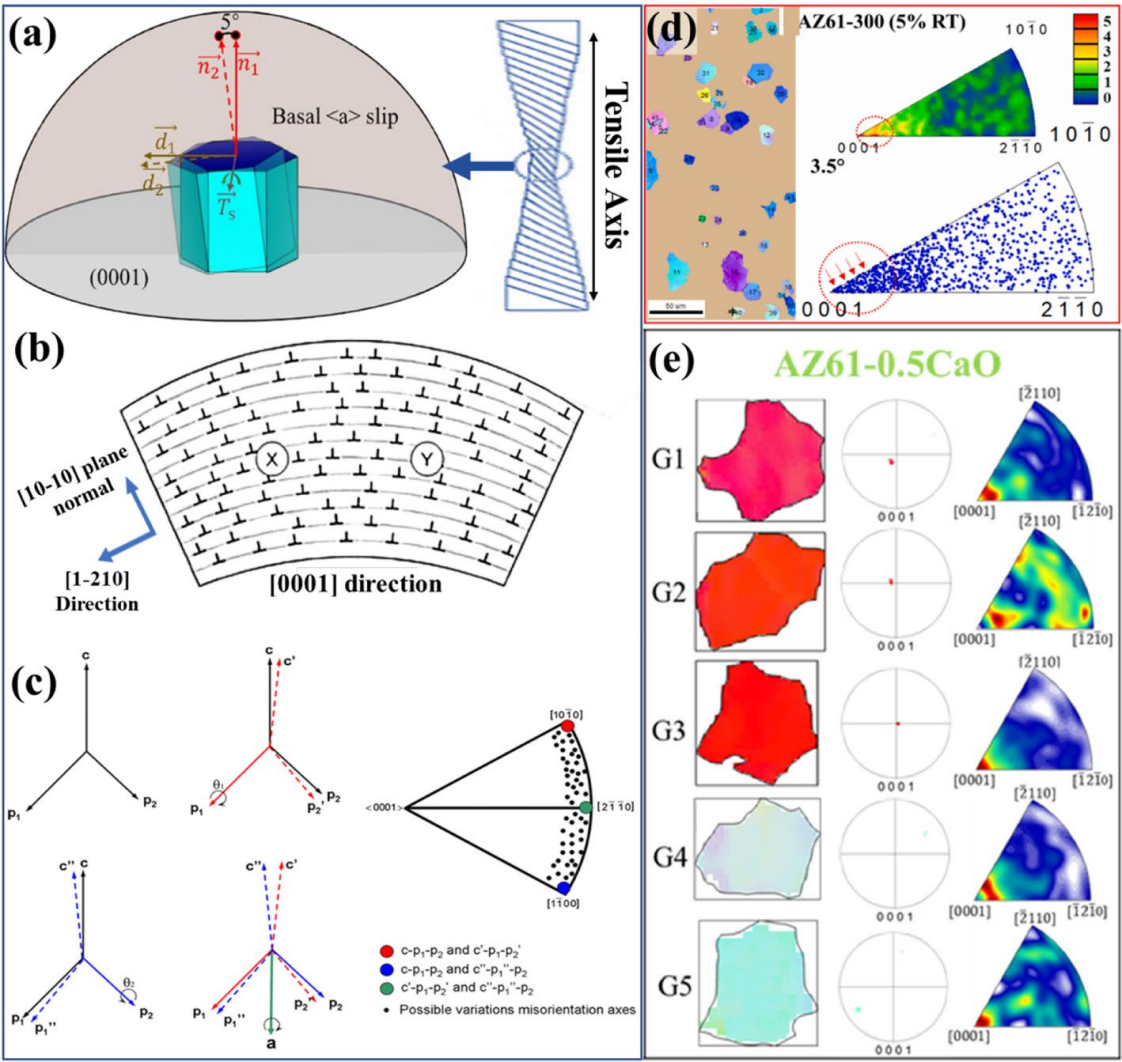
**a** Schematic illustration for the lattice rotation mechanism during deformation, where $\underset{{d}_{1}}{\to }$, $\underset{{d}_{2}}{\to }$, $\underset{{n}_{1}}{\to }$, $\underset{{n}_{2}}{\to }$ and $\underset{{T}_{s}}{\to }$ are the initial slip shear direction, the post-deform slip shear direction, the initial vector of c-axis, the post-deform vector of c-axis and the Taylor axis, respectively (Yang et al. 2023). **b** Schematic of a bent single crystal with homogeneously distributed edge dislocations from prismatic < a > slip (b = 1/3[1–210]) on (10–10) planes. The crystal bends about [0001], causing neighboring EBSD points X and Y to be misoriented around this axis. **c** Schematics of hcp lattices showing possible IGMAs formed by lattice rotations about the < 10–10 > axis. The resultant misorientation axis between rotated lattices is parallel to the a-axis, with possible axes plotted on an equal-angle projection (Chun et al. 2010). **d** An example showing IGMA analysis of 5% deformed AZ61 Mg alloy extruded at 300 °C (Tariq et al. 2024b). **e** Another example showing IGMA analysis on the 5 grains picked from a 10% deformed AZ61-1CaO alloy (Here different grain colors represent different crystallographic orientations) (Chaudry et al. 2024c)

During plastic deformation, each slip system produces a characteristic pattern of lattice rotation, which can be described by a specific crystallographic rotation axis often referred to as the Taylor axis (Winther 2008). For any slip system, this axis lies within the corresponding slip plane and remains orthogonal to the slip direction. Mathematically, it can be determined through the vector product of the slip-plane normal and the slip direction as follows (Fig. 3a);

4 $${\mathbf T}_{\mathbf s}\boldsymbol\;\boldsymbol=\boldsymbol\;{\mathbf n}_{\mathbf s}\boldsymbol\;\times\boldsymbol\;{\mathbf d}_{\mathbf s}$$

Here, *T*_*s*_, *n*_*s*_ and d_*s*_ denote the Taylor axis, slip plane normal, and slip direction for a given slip system, respectively. In EBSD-based deformation analysis, this concept forms the basis of the In-grain misorientation axis (IGMA) approach. IGMA identifies which slip systems were activated by comparing the experimentally measured lattice-rotation axis inside a grain with the theoretically computed Taylor axis of possible slip systems. The use of IGMA relies on several key premises: (1) orientation gradients observed within a deformed grain originate from the curvature of the crystal lattice caused by dislocation glide, (2) this curvature can be represented as a rotation of the lattice around the Taylor axis associated with the active slip system, and (3) the Taylor axis corresponds to a low-index direction that lies in the slip plane and is perpendicular to the slip direction.

The deformation substructure that develops within a single crystal undergoing plastic strain is schematically represented in Fig. 3b. When the dislocation population is assumed to arise predominantly from prismatic < a > slip, the resulting dislocations are expected to be of pure edge character. Under this condition, the crystal should experience lattice bending about an axis parallel to the line direction of these edge dislocations. Because prismatic < a > slip on {10–10} plane produces edge dislocations with a Burgers vector of 1/3[1–210], their line direction is aligned with the crystallographic [0001] direction (Anderson et al. 2017). Consequently, two neighboring points X and Y, representing adjacent measurement nodes in the EBSD grid for IGMA evaluation, should exhibit a finite misorientation about the [0001] axis, with the magnitude of this axis-angle pair scaling proportionally with the local geometrically necessary dislocation density. The occasional activity of secondary slip modes can introduce a limited number of additional dislocations whose line vectors deviate from [0001]; these contributions slightly distort the local lattice curvature, thereby broadening the IGMA intensity distribution around the ideal (0001) axis rather than producing a distinctly different orientation clustering. Since the {10–10} Taylor axis corresponding to the {11–22} slip system has no < c > component, this slip mode produces lattice rotations that align the IGMA predominantly along {uvt0} directions, as shown in Fig. 3c. A single variant of {11–22} slip system activity can cause lattice rotation around one type of Taylor axis. For example, p_1,_ which shows [10-10] axis or p_2_ shows [1–100] axis. In the other case, if more than 2 variants of {11–22} slip systems are active, the misorientation axis can deviate from its ideal Taylor axis. It can also occur when adjacent subgrains have undergone different slip‑induced lattice rotation paths, so that the misorientation between them does not correspond to a single ideal Taylor axis. This mechanism is demonstrated in the lower panels of Fig. 3(c), where two lattices, each having rotated by an equal angle but about distinct Taylor axis (e.g., p_1_ and p_2_), can generate a net misorientation with the axis close to a = [2-1-10] axis. Importantly, the actual misorientation axis depends on the sequence, axis, angle, and direction of prior lattice rotations. As a result, the observed misorientation axis can form a continuous distribution between p₁ and p₂, as schematically represented by black dots along the edge of the unit triangle.

EBSD serves as a powerful tool for quantifying IGMA across a statistically meaningful population of grains, enabling a robust assessment of slip activity in polycrystalline hcp metals. Because EBSD provides orientation information at high spatial resolution, the resulting IGMA maps allow deformation-induced lattice rotations to be characterized with far greater accuracy than is achievable through single-grain or single-point measurements. In practical terms, the IGMA distribution for an individual deformed grain is obtained through the following sequential steps: (i) identifying the grain of interest, (ii) collecting all orientation data from the scanning grid points contained within that grain, (iii) calculating the misorientation axis for every pair of neighboring grid points, and (iv) projecting the resulting axis-density distribution, expressed in multiples of a uniform distribution (mud), onto the standard unit triangle using an equal-angle scheme. According to established criteria, grains exhibiting a maximum IGMA intensity greater than 2 mud are classified as having a pronounced, non-random IGMA, whereas grains with intensities below this threshold are regarded as displaying a more uniform, non-preferential IGMA pattern (Chun et al. 2010; Ando and Tonda 2000). The IGMA analyses can distinguish between slip modes across a misorientation range of 2.5–5° (Ha et al. 2021). Within this range, in-grain misorientation primarily increases due to the activation of slip systems. To simplify the explanation of the correlation between the activated slip system and IGMA, the activation of a single slip in a crystal is considered. Building on the above methodological basis, the practical utility of IGMA becomes evident when applied to specific deformation cases. For instance, in the study of AZ61-300 Mg alloy deformed to 5% strain at room temperature, representative grains were extracted from the IPF maps (Fig. 3d) (Tariq et al. 2024b). When these grains were subjected to IGMA analysis, their misorientation-axis distributions exhibited a clear clustering around the [0001] pole. This indicated that prismatic slip was the predominant deformation mode in the AZ61-300 alloy at room temperature. A similar interpretation can be extended to the alloy series investigated by Chaudry et al., where AZ61-1CaO samples were deformed to 10% strain at room temperature (Fig. 3e). The IGMA maps generated from representative grains (G1-G5) reveal that the alloy exhibits stronger IGMA intensities around the [0001] rotation axis. This confirms prismatic < a > slip as the principal deformation mechanism (Chaudry et al. 2024c).

Despite its utility, IGMA characterization using EBSD must be applied with care due to the finite angular resolution inherent to the technique. Accurate interpretation requires the selection of a minimum reliable misorientation angle to avoid artifacts arising from measurement noise. This threshold is typically established by examining the statistical behavior of neighboring-point misorientations within a representative grain and tracking both the fraction of point pairs contributing to each misorientation level and the corresponding accumulated fraction. Through this calibration procedure, misorientation angles below the resolution limit can be excluded, ensuring that only physically meaningful lattice rotations contribute to the IGMA analysis.

## Slip trace and slip transfer analysis

**Fig. 4:**
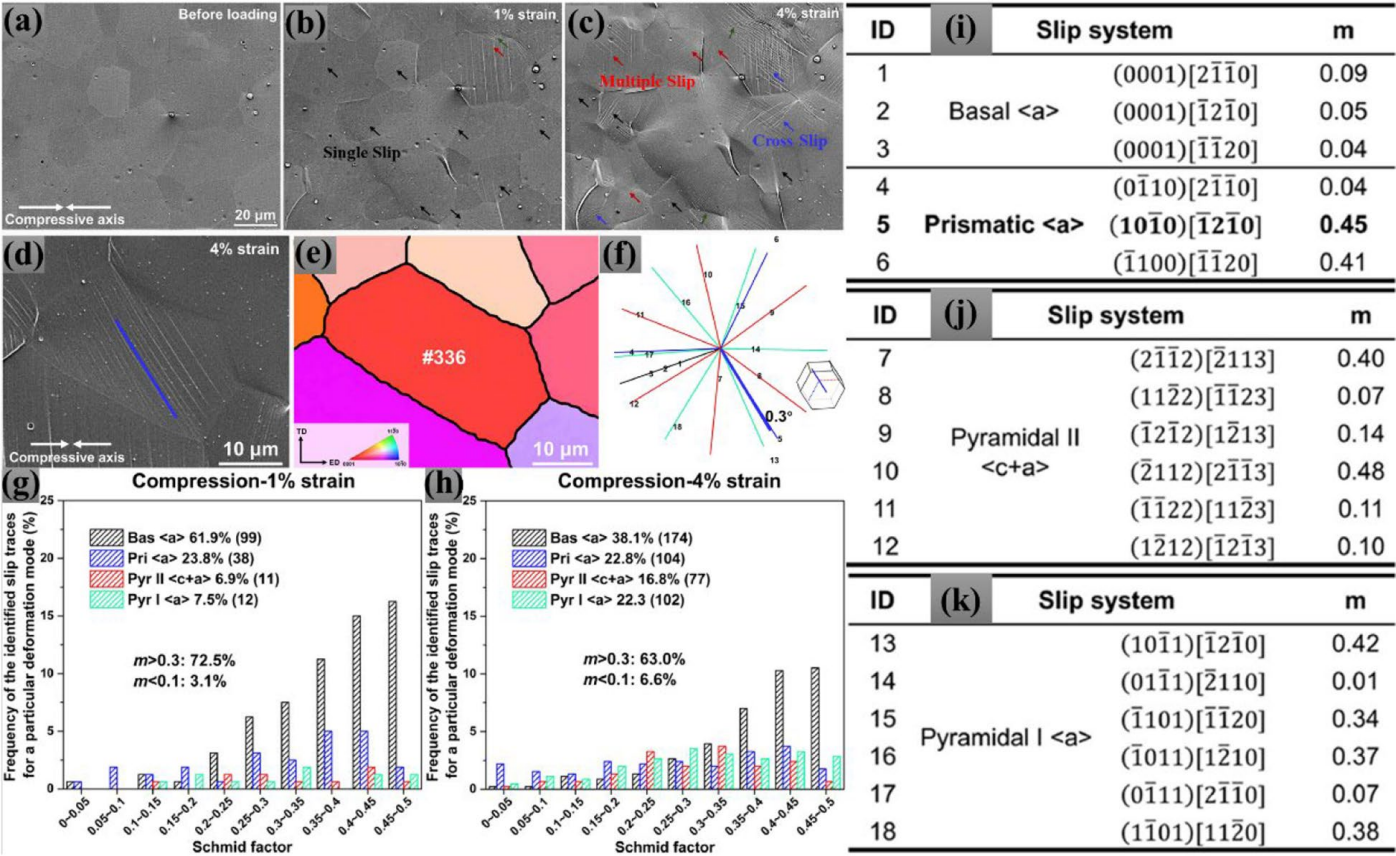
Secondary Electron (SE) SEM micrographs and analysis showing the microstructural evolution and slip activity in an aged Mg–10Y sheet under compression. **a** Microstructure before loading. **b** After 1% compressive strain and (**c**) After 4% compressive strain. **d-f** Methodology for slip-trace identification demonstrated for representative grain number 336 at 4% strain: (**d**) SEM image, (**e**) corresponding inverse pole figure (IPF) map before loading, and (**f**) calculated slip-trace orientations based on crystallographic geometry. **g**, **h** Frequency distribution of Schmid factors (m) for different slip systems after (**g**) 1% and (**h**) 4% compressive strain. **i-k** Calculated Schmid factors (m) for individual slip systems (Ni et al. 2024)

Slip trace analysis is a powerful crystallographic approach employed to quantitatively determine the active deformation mechanisms in hexagonal close-packed (HCP) metallic systems such as Mg alloys. The technique combines high-resolution secondary electron (SE) imaging with EBSD data to correlate surface deformation features with the underlying crystallographic orientation of individual grains (Yuan et al. 2022). When plastic deformation occurs, dislocations glide along specific slip planes and directions, producing discrete surface steps, referred to as slip traces, that directly reflect the activation of certain slip systems. Many studies have identified slip systems in Mg by comparing the slip trace observed in SEM with the possible slip directions calculated using Euler angles obtained in EBSD (Xu et al. 2019). In practical implementation, EBSD maps are first collected on the undeformed or lightly deformed specimen to acquire Euler angles (ϕ₁, Φ, ϕ₂) for each grain. Using these orientations, theoretical slip trace directions are calculated for all potential slip systems (basal < a >, prismatic < a >, and pyramidal < c + a > by projecting crystallographic vectors onto the specimen surface through rotation matrices (Vermeij et al. 2023). The experimentally measured trace orientations, obtained from SE–SEM images after deformation, are then quantitatively compared with these theoretical traces. The slip system corresponding to the smallest angular deviation is identified as the active system. To strengthen this identification, the Schmid factor is also calculated. Systems exhibiting both high m-values (typically > 0.3) and low deviation angles (< 5°) are taken as the dominant deformation modes. For instance, Ni et al. recently performed quasi-in-situ compression tests to investigate the microstructural evolution of the same region at different strains, as shown in Fig. 4 (a-c). When the alloy was strained from an undeformed (Fig. 4a) to a deformed state (1%, Fig. 4b and 4%, Fig. 4c), numerous slip traces were observed with a thicker and sparser morphology. When compressed to 4% strain, the preceding traces exhibited a further intensification, accompanied by an increase in the frequency of multiple slip and cross-slip events.

The average Euler angle of each grain was obtained by EBSD, based on which the theoretical slip traces for each grain can be calculated. Comparing the theoretical slip trace with the experimental slip trace, the activated slip system can be obtained. Slip trace analysis was implemented as the following steps, taking grain number 336 as an example (Fig. 4). Firstly, the experimental slip trace was obtained in the SEM image (Fig. 4d). Secondly, all the possible theoretical slip traces were obtained (Fig. 4f) using a homemade MATLAB code, based on the Euler angle from the undeformed EBSD (Fig. 4e). Finally, the slip system corresponding to the theoretical slip trace with the smallest deviation angle to the experimental slip trace was deemed to be the activated one, and its m was obtained according to Fig. 4(i-k). For grain 336, the active slip system was identified as a prismatic slip system, with an m value of 0.45 and an angular deviation of 0.3°. This methodology was successfully employed for the precise identification of all the non-basal slip systems. However, since the three basal slip variants have a single slip plane, they cannot be distinguished by slip trace analysis. In such cases, the activated slip system was assumed to be the one with the highest m value.

Further investigation of the m-value distributions (Fig. 4 (g-h)) for both 1% and 4% compression reveals that the majority of the activated slip systems—exceeding 60%—exhibited relatively higher than 0.3 m-values. Only a small fraction, less than 10%, showed low m-values (< 0.1). Notably, this trend remained consistent regardless of the applied deformation level or strain, indicating that the overall m-value characteristics are largely insensitive to the specific loading strain. They further calculated the normalized m-value factor to obtain the relationship between slip activation and Schmid law by using the following equation (Ni et al. 2024);

5 $${\mathbf m}_{\mathbf{normal}}\boldsymbol\;\boldsymbol=\boldsymbol\;\frac{\boldsymbol m\boldsymbol-{\boldsymbol m}_{\boldsymbol m\boldsymbol i\boldsymbol n}}{\mathbf2({\mathbf m}_{\boldsymbol m\boldsymbol a\boldsymbol x}\boldsymbol-{\mathbf m}_{\mathbf{min})}}$$

They reported that the m_normal_ distribution under compression followed a trend similar to that of m. Most m_normal_ values were greater than 0.3, while only a small portion fell below 0.1. At 4% compression strain, the fraction of slip systems exhibiting low m_normal_ values (< 0.1) increased to 10.6%, suggesting that more slip systems departed from Schmid-type activation as deformation progressed. Although slip activity was predominantly consistent with the Schmid law, a notable subset of systems did not conform to it, likely due to discrepancies between the local stress fields and the applied macroscopic stress during compression.

While slip trace analysis can directly identify the active slip plane, distinguishing the exact slip direction within that plane often requires supplementary methods, as multiple directions may exist within the same plane. More advanced methods, such as those combining EBSD with high-resolution digital image correlation (HR-DIC), can provide more detailed information on the slip direction and dislocation characteristics.

**Fig. 5:**
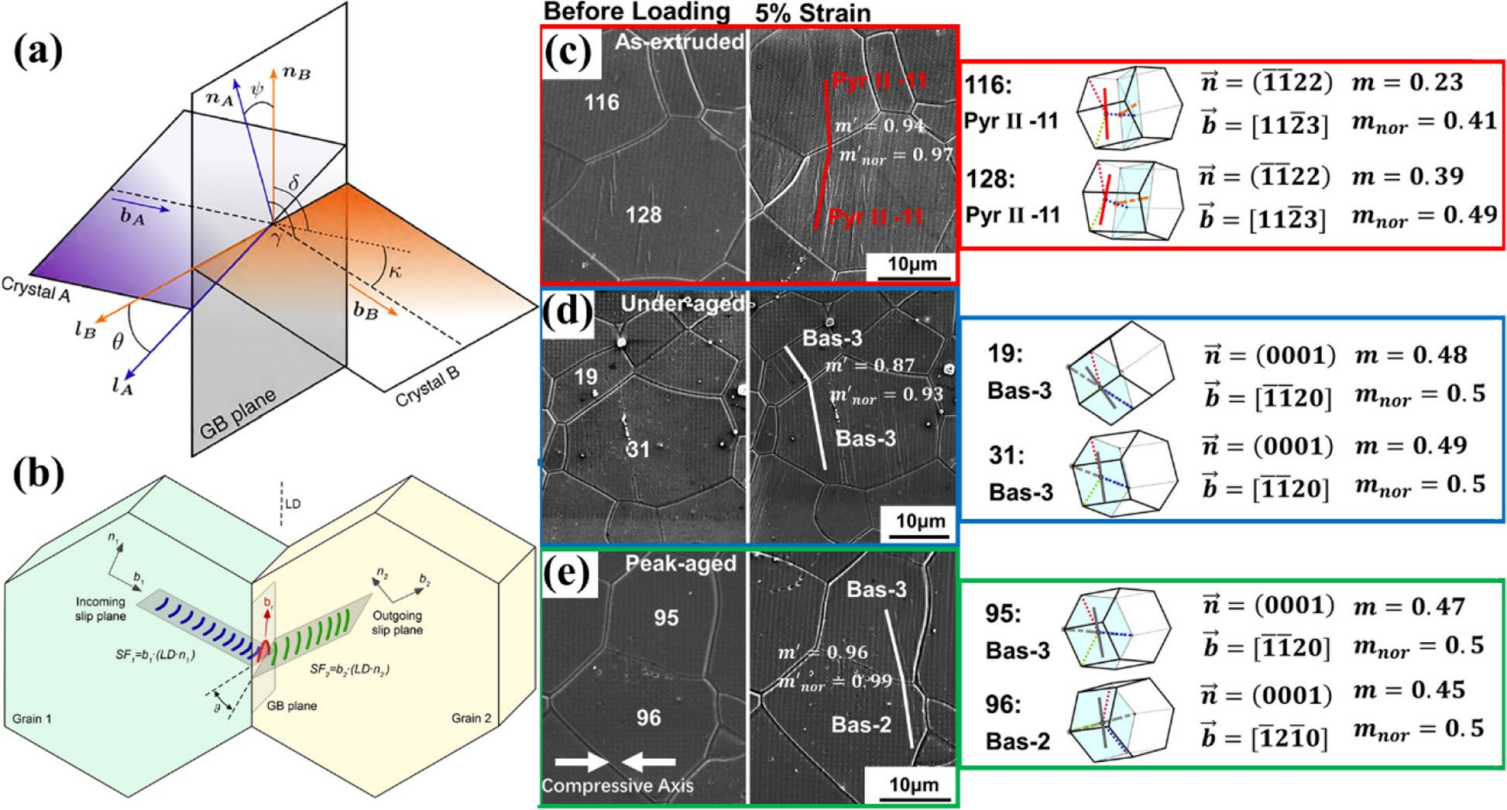
**a** Geometrical criteria for slip transfer/blocking located at the grain boundary. Here $\underset{{b}_{A}}{\to }$ and $\underset{{n}_{A}}{\to }$ are the Burgers vector and the slip plane normal of the incoming slip plane of crystal A. $\underset{{b}_{B}}{\to }$ and $\underset{{n}_{B}}{\to }$ are the Burgers vector and the slip plane normal of the incoming slip plane of crystal A. *κ* is the angle between the Burgers vectors of the incoming and outgoing slip systems. *Ψ* is the angle between the slip planes normal and *θ* is the angle between the traces of the incoming and outgoing slip planes with the GB plane (Li et al. 2025). **b** Schematic of slip transmission: dislocations from an incoming slip system in grain A propagate through the grain boundary AB onto the outgoing slip system in grain B. The transmission of each dislocation produces a residual grain boundary defect. **c-e** Sequential SE SEM photomicrographs showing representative examples of slip transfer at GB for as-extruded, under-aged and peak-aged samples, respectively. The right-hand unit cell visualizes the identified active slip systems (Ni et al. 2022)

Following the determination of slip activity, the next critical step is to assess slip interactions at grain boundaries, which play a critical role in controlling strain evolution within the microstructure. In particular, the ability of dislocation slips to either transfer across a grain boundary (GB) or become blocked at GB is a key factor controlling how deformation accumulates within polycrystalline materials. Slip transfer tends to promote more uniform deformation, whereas slip blocking can lead to localized strain, elevated dislocation pile-ups, and the early onset of damage or cracking (Zhang et al. 2022). To evaluate whether an active slip system in one grain is likely to transmit into its neighboring grain, several geometrical descriptors have been introduced in the literature (Hua et al. 2021; Chai et al. 2021). These criteria assess how well the crystallography of the incoming slip system aligns with that of the potential outgoing system across the boundary. As illustrated in Fig. 5 (a, b), such assessments typically consider parameters reflecting the relative orientations of slip planes, slip directions, and their intersection with the GB (Li et al. 2025). These criteria typically involve several geometric parameters, including the angle κ between the Burgers vectors of the incoming ($\underset{{b}_{A}}{\to }$) and outgoing ($\underset{{b}_{B}}{\to }$) slip systems*,* the angle *ψ* etween their respective slip-plane normal ($\underset{{n}_{A}}{\to }$ and $\underset{{n}_{B}}{\to }$), and the angle *θ* between the slip-plane traces on the grain boundary, often known as the twist angle. Among the most widely applied measures are the Luster-Morris parameter (m′) and the LRB criterion, which are defined as follows (Shen et al. 1988; Luster and Morris 1995):

The increase in Luster-Morris parameter (*m′*) values predicts the ease of slip transfer geometrically (Jiang et al. 2020). Previous studies have focused on evaluating the effectiveness of *m′* for predicting whether slip or twinning events will transmit across or be blocked at GBs (Liu et al. 2016; Guo et al. 2014). This emphasis largely stems from the fact that all parameters required for calculating *m′* can be directly obtained from slip-trace observations and surface EBSD measurements. In contrast, the use of the LRB criterion has been debated, primarily because its formulation requires knowledge of the twist angle θ, which depends on the three-dimensional orientation of the GB plane—information that is generally inaccessible when only surface data are available (Ni et al. 2022). Although the LRB approach incorporates both the respective Burgers vectors and the coplanarity of the interacting slip systems through their twist relationship, its predictive capability has shown inconsistencies (Li et al. 2025; Genée et al. 2017). Several studies have indicated that the LRB criterion may perform poorly in materials where deformation is governed by particular slip modes or in situations involving complex boundary geometries or multiple activated slip systems (Genée et al. 2017; Chen et al. 2023).

Given that basal slip possesses the lowest CRSS among the other deformation modes in Mg alloys, it is typically the first and most easily activated slip mechanism. Consequently, the interaction between basal dislocations and grain boundaries becomes a dominant factor governing grain-boundary strengthening (Hall–Petch effect) as well as the macroscopic mechanical behavior of Mg alloys (Xing et al. 2022). While basal slip can occasionally transmit through suitably oriented grain boundaries, facilitating localized deformation, GBs more commonly impede basal dislocation motion, which leads to elevated stress concentrations in the vicinity of the boundary (Tariq et al. 2024e). These localized stresses can subsequently trigger the activation of higher-CRSS deformation modes, such as prismatic or pyramidal slip, or even extension twinning. The activation of these additional mechanisms contributes to a more distributed and homogeneous deformation response (Shi et al. 2023). For instance, Ni et al. performed quasi-in-situ grain-by-grain slip transfer analysis using EBSD on the as-extruded, under-aged, and peak-aged Mg-10Y alloy sheets as shown in Fig. 5 (c-e) (Ni et al. 2022). They also calculated the normalized *m′* values using Eq. 5, where a value approaching unity indicates that the measured *m′* is close to its theoretical maximum. In the as-extruded condition, the grain pair 116–128 (Fig. 6c) exhibited a clear slip transfer event, with both slip systems identified as pyramidal II. Although the Schmid factor (*m*) of the activated slip in grain 116 was relatively low (0.23), both transferred slip systems showed high normalized *m* values (> 0.3). Furthermore, the Luster–Morris parameters (*m′* and *m′ₙₒᵣₘₐₗ*) exceeded 0.9, indicating strong geometric compatibility between the transferred slip systems. A similar behavior was observed in the under-aged and peak-aged conditions (Fig. 6 d and 6e), where all participating transferred slip systems exhibited high *m′* and *m′ₙₒᵣₘₐₗ* values (> 0.85), confirming the consistency of slip transfer across different aging states.

A key limitation of conventional EBSD-based slip transfer analysis is its inherently two-dimensional nature, which restricts characterization to surface observations and prevents accurate reconstruction of the true three-dimensional geometry of grain boundaries (McDonald et al. 2015). Because slip transfer and blocking are strongly influenced by out-of-plane boundary orientation, curvature, and grain morphology, relying solely on surface data can lead to missing or inaccurate interpretations of intergranular deformation. To overcome these limitations, advanced 3D characterization techniques such as diffraction contrast tomography (DCT) or synchrotron-based 3D X-ray microscopy provide spatially resolved information on grain shape, orientation, and boundary topology, enabling far more reliable assessment of slip interaction mechanisms across grain boundaries.

## Conclusions and future outlooks

This review emphasizes the critical contributions of EBSD-based techniques in interpreting slip activity, slip transfer, and intergranular deformation mechanisms in Mg alloys. Schmid factor and in-grain misorientation axis (IGMA) analyses provide a quantitative framework for understanding the activation of basal, prismatic, and pyramidal slip systems, revealing how grain orientation, crystallographic texture, and local stress states control plastic deformation. Slip trace analysis extends these approaches by directly correlating surface features with crystallographic orientation to identify active slip planes and, in combination with Schmid factor assessment, the dominant deformation modes. Despite their utility, EBSD-based methods are inherently limited by their two-dimensional nature, which restricts characterization of true grain-boundary geometry and three-dimensional slip transfer behavior. Advanced methods that have shown significant promise in addressing these limitations include high-resolution digital image correlation and three-dimensional characterization techniques such as diffraction contrast tomography, which represent promising directions for future research.

Further development of the integrated experimental and computational approach is required for the future. Linking 3D EBSD/DCT datasets with crystal plasticity finite element modeling can provide predictive insights into local stress states, slip compatibility, and strain evolution across grain boundaries. The extension of these methodologies to high-strain-rate or cryogenic deformation regimes offers new opportunities to understand the interplay between basal and non-basal slip systems under extreme conditions and gives a basis for designing Mg alloys with improved ductility and strength. In all, combining multiscale, three-dimensional experimental techniques with robust modeling frameworks represents an important pathway toward further progress in our understanding of plasticity in HCP metals and the rational engineering of next-generation lightweight alloys.

## Data Availability

The dataset used during the current study are available from the corresponding author on reasonable request.
